# Effects of alcohol consumption, ALDH2 rs671 polymorphism, and Helicobacter pylori infection on the gastric cancer risk in a Korean population

**DOI:** 10.18632/oncotarget.14250

**Published:** 2016-12-21

**Authors:** Sarah Yang, Jeonghee Lee, Il Ju Choi, Young Woo Kim, Keun Won Ryu, Joohon Sung, Jeongseon Kim

**Affiliations:** ^1^ Molecular Epidemiology Branch, Division of Cancer Epidemiology and Prevention, National Cancer Center, Goyang, Korea; ^2^ Complex Disease & Genome Epidemiology Branch, Department of Public Health, Graduate School of Public Health, Seoul National University, Seoul, Korea; ^3^ Center for Gastric Cancer, National Cancer Center Hospital, National Cancer Center, Goyang, Korea; ^4^ Department of Cancer Control and Policy, Graduate School of Cancer Science and Policy, National Cancer Center, Goyang, Korea

**Keywords:** gastric cancer, alcohol, ALDH2 rs671 polymorphism, helicobacter pylori infection, interaction

## Abstract

The effect of alcohol consumption on the risk of gastric cancer (GC) has not yet been fully elucidated, and an aldehyde dehydrogenase 2 (*ALDH2*) polymorphism, rs671, is a genetic variant that influences alcohol consumption in East Asians. Additionally, the discrepancy between the *Helicobacter pylori* (*H. pylori*) infection prevalence and GC incidence across Asian countries has not been explained. This study evaluated the effects of alcohol consumption and genetic susceptibility to defective acetaldehyde metabolism on the GC risk and their interactions with *H. pylori* infection. This study included 450 Korean GC cases and 1,050 controls recruited at the National Cancer Center. Data for 795 patients and 4,893 controls were used for further confirmation of the effect of rs671. Increased GC risks were evident for rs671 A allele carriers (odds ratio (OR), 1.23; 95% confidence interval (CI), 1.08-1.41) and *H. pylori*-infected individuals (OR, 7.07; 95% CI, 4.60-10.86), but no dose-response association with alcohol consumption was observed. Furthermore, the interactions between these factors were not significant. This study has demonstrated that alcohol consumption and rs671 should be considered simultaneously when assessing the GC risk. Additionally, alcohol-related factors were not found to interact with *H. pylori* infection, and further studies evaluating other environmental factors are required to explain the Asian enigma.

## INTRODUCTION

Excessive drinking of alcoholic beverages has been reported to affect various cancers, including liver, breast, colorectal, oral cavity, and esophageal cancers [1–9]. However, there are some discrepancies among studies evaluating the carcinogenic effect of alcohol on gastric cancer [2, 10–12]. These differences may be due to various confounding factors, such as age, sex, cigarette smoking, socio-economic status, ethnicity, and genetic susceptibility, which affect both disease development and alcohol consumption behaviors [13].

Acetaldehyde, which is a metabolite of alcohol, is known to increase the cancer risk, and the aldehyde dehydrogenase 2 (*ALDH2*) gene encodes an enzyme that detoxifies acetaldehyde to acetate. However, a functional single nucleotide polymorphism (SNP) in the *ALDH2* gene, rs671, which is abundant in the East Asian population, causes formation of an inactive subunit of the enzyme, resulting in an inability to eliminate acetaldehyde, thereby causing adverse effects following alcohol intake [14]. Consequently, the blood acetaldehyde level has been reported to be higher in AG heterozygotes or AA homozygotes compared to GG homozygotes after alcohol consumption [15]. The polymorphic A allele of rs671 is known to have a significant effect on the amount of alcohol consumed [16–19], and therefore, it is important to consider the genotype when assessing the risk of gastric cancer related to alcohol intake. Several studies including East Asian populations [17, 20–23] have demonstrated that *ALDH2* polymorphisms, especially rs671, are associated with an increased risk of gastric cancer, and studies from Korea [17] and Japan [20] have reported a possible interaction between alcohol consumption and the *ALDH2* genotype on the incidence of gastric cancer. However, categorization of the alcohol consuming groups, which exhibited significant interactions with the *ALDH2* genotype, differed between the two studies, and further validation is required to define and understand the mechanism underlying this relationship.

In 2012, gastric cancer was reported to be the third leading cause of cancer death worldwide, and the East Asian population contributed to more than half of the total incidence and had the highest mortality rate [24]. A recent report of cancer statistics in Korea has revealed that the cancer with the highest incidence among men is gastric cancer, which is also the fourth most common cancer among women [25]. This phenomenon could be due to the presence of the rs671 polymorphism in the East Asian population [26], along with the high prevalence of *Helicobacter pylori* (*H. pylori*) infection, which is a well-known cause of gastric cancer [27–29]. However, the discrepancy between the high *H. pylori* seroprevalences and the different gastric cancer incidence rates across Asian populations [30] has been found to be not solely attributed by the presence of different bacterial strain types. Hence, assessment of the interactions of *H. pylori* with other environmental factors has been emphasized [28, 31] to better understand the differences between the anticipated and observed gastric cancer incidences. From this perspective, we hypothesized that an East Asian-specific interaction between *H. pylori* infection and alcohol consumption on the gastric cancer risk may occur in this region where the *ALDH2* rs671 polymorphism is prevalent, thereby causing the excessive gastric cancer incidence. Further, *H. pylori* infection induces chronic gastric inflammation, and acetaldehyde accumulation causes oxidative stress and inflammation, and these factors may act in concert in the development of gastric cancer. A recent Korean case-cohort study has reported that only individuals without *H. pylori* infection who are heavy drinkers exhibit an increased gastric cancer risk [32]. However, consideration of the *ALDH2* genotype is essential in further evaluations of the relationship between alcohol consumption and *H. pylori* infection and their joint effect on the gastric cancer risk.

In this study, we investigated the roles of alcohol consumption, the *ALDH2* rs671 polymorphism, and their interaction in the risk of gastric cancer, with comparison of the differences in results between the sexes. In addition, we hypothesized that an interaction of *H. pylori* infection with alcohol drinking behavior, as well as with genetic susceptibility to defective acetaldehyde metabolism, may underlie the complex etiology of gastric cancer.

## RESULTS

### Demographic characteristics of the study subjects

The general characteristics of the study participants from the National Cancer Center (NCC) are described in Table 1. Only the male cases and controls showed significant differences in alcohol intake, composition of alcohol drinking groups, and smoking statuses. Distribution of the rs671 genotype did not differ between the cases and controls, and there were more individuals with over 12 years of education and a higher household income among the controls than among the cases for both sexes. The demographic characteristics and average daily alcohol intake of the Korean Genome Epidemiology Study (KoGES) participants are shown in Supplementary Table 1. There were no differences in the potential confounders for different rs671 genotypes among the control samples, and the average alcohol intake and alcohol drinking status were significantly associated with this polymorphism (Supplementary Table 2). Among the controls in the different drinking groups, age, sex, smoking status, education level, income level, and fruit/vegetable intake significantly differed, reflecting the effects of the various confounding factors on alcohol consumption behaviors (Supplementary Table 3).

**Table 1:** Characteristics of the National Cancer Center (NCC) subjects, stratified by disease status and sex

	Total					Men					Women				
	Case (n=450)		Control (n=1050)		P value†	Case (n=297)		Control (n=492)		P value†	Case (n=153)		Control (n=558)		P value†
	n	Mean ±S.D	n	Mean ±S.D		n	Mean ±S.D	n	Mean ±S.D		n	Mean ±S.D	n	Mean ±S.D	
**Age (years)**	450	55.40±10.74	1050	52.04±8.52	<.0001	297	56.58±10.08	492	54.1±8.73	0.001	153	53.12±11.61	558	50.23±7.91	<0.0001
**ALDH2 genotype (rs671)**					0.19					0.54					0.47
**A/A**		5(1.11)		22(2.10)			4(1.35)		10(2.03)			1(0.65)		12(2.15)	
**A/G**		141(31.33)		292(27.81)			103(34.68)		155(31.50)			38(24.84)		137(24.55)	
**G/G**		304(67.56)		736(70.10)			190(63.97)		327(66.46)			114(74.51)		409(73.30)	
**Alcohol intake (g/day), only among drinkers**	289	26.70±32.07	687	14.61±19.73	<.0001	224	32.34±33.96	405	20.81±22.64	<0.0001	65	7.26±10.72	282	5.70±8.86	0.28
**Alcohol drinking status by amount (%)**					<.0001					<0.0001					0.36
**Never/Rare drinkers (<3 g/day)**		183(44.85)		578(55.05)			73(27.34)		148(30.08)			110(78.01)		430(77.06)	
**Light drinkers (3-20 g/day)**		90(22.06)		298(28.38)			66(24.72)		186(37.80)			24(17.02)		112(20.07)	
**Heavy drinkers (≥20 g/day)**		135(33.09)		174(16.57)			128(47.94)		158(32.11)			7(4.96)		16(2.87)	
**Smoking status (%)**					<.0001					0.002					0.57
**Non-smoker**		170(39.26)		614(58.48)			39(13.54)		96(19.51)			131(90.34)		518(92.83)	
**Ex-smoker**		132(30.48)		262(24.95)			125(43.40)		244(49.59)			7(4.83)		18(3.23)	
**Current smoker**		131(30.25)		174(16.57)			124(43.06)		152(30.89)			7(4.83)		22(3.94)	
**Education level (%)**					<.0001					<0.0001					<0.0001
**<12 years**		153(35.42)		141(14.21)			99(34.49)		57(12.45)			54(37.24)		84(15.73)	
**≥12 years**		279(64.58)		851(85.79)			188(65.51)		401(87.55)			91(62.76)		450(84.27)	
**Household income -10,000 KRW/month (%)**					<0.0001					<0.0001					0.001
**Below 200**		151(39.22)		175(18.8)			103(40.08)		70(15.98)			48(37.50)		155(21.30)	
**200-400**		138(35.84)		424(45.54)			99(38.52)		213(48.63)			39(30.47)		211(42.80)	
**Above 400**		96(24.94)		332(35.66)			55(21.40)		155(35.39)			41(32.03)		177(35.90)	
**Fruit and vegetable intake (%)a,b**					<.0001					0.41					0.0012
**Lowest tertile (<426.55 g/day)**		193(46.06)		344(33.43)			140(50.72)		224(46.09)			53(37.06)		120(22.10)	
**Middle tertile (<649.47 g/day)**		124(29.59)		341(33.14)			82(29.71)		151(31.07)			42(29.37)		190(34.99)	
**Highest tertile (≥649.47 g/day)**		102(24.34)		344(33.43)			54(19.57)		111(22.84)			48(33.57)		233(42.91)	
**H. pylori infection status (%)**					<.0001					<0.0001					<0.0001
**Positive**		412(91.56)		639(60.86)			272(91.58)		324(65.85)			140(91.50)		315(56.45)	
**Negative**		38(8.44)		411(39.14)			25(8.42)		168(34.15)			13(8.50)		243(43.55)	
**Family history of gastric cancer in 1st degree relatives (%)**					0.0002					0.004					0.11
**Yes**		93(20.67)		138(13.14)			67(22.56)		71(14.43)			26(16.99)		67(12.01)	
**No**		357(79.22)		912(86.86)			230(77.44)		421(85.57)			127(83.01)		491(87.99)	

### Independent associations of gastric cancer risk with alcohol consumption, *ALDH2* polymorphism, and *H. pylori* infection

When the subjects were classified into alcohol-consuming groups by drinking dose, the odds ratio (OR) for the total participants was 1.83 (95% confidence interval (CI), 1.31-2.54) and that for the male participants was 1.78 (95% CI, 1.23-2.58) for the heavy drinkers compared with the never/rare drinkers in the minimally adjusted model (Table 2). However, no significant risk was observed following full adjustments for potential confounders, and the female subjects did not show any increase in the gastric cancer risk in association with alcohol consumption. The individuals with *H. pylori* infection had a 7.07-fold higher risk compared to those with no infection (fully adjusted, 95% CI, 4.60-10.86), and the OR for the females (7.54; 95% CI, 3.91-14.55) was slightly higher than that for the males (7.40; 95% CI, 4.11-13.31). The individuals with the A allele had an evidently increased risk of gastric cancer compared to the GG homozygotes. The OR for the total participants with the AA/AG genotype was 1.23 (95% CI, 1.08-1.41), and that for the male participants was 1.33 (95% CI, 1.13-1.58). However, this trend was not observed among the female participants.

**Table 2:** Association between alcohol drinking status, *H. pylori* infection, and *ALDH2* rs671 polymorphism and gastric cancer risk, stratified by sex

	Case (%)	Control (%)	Minimally Adjusted ^a^		Fully Adjusted	
			OR(95% CI)	P value *	OR(95% CI)	P value*
***Alcohol intake***						
Total						
Never/Rare drinkers (<3 g/day)	183(44.85)	578(55.05)	1.00(reference)	0.001	1.00(reference)^b^	0.24
Light drinkers (3-20 g/day)	90(22.06)	298(28.38)	0.81(0.59-1.10)		0.77(0.53-1.11)	
Heavy drinkers (≥20 g/day)	135(33.09)	174(16.57)	**1.83(1.31-2.54)**		1.33(0.90-1.97)	
Men						
Never/Rare drinkers (<3 g/day)	73(27.34)	148(30.08)	1.00(reference)	0.001	1.00(reference)^b^	0.06
Light drinkers (3-20 g/day)	66(24.72)	186(37.80)	0.75(0.50-1.12)		0.78(0.48-1.27)	
Heavy drinkers (≥20 g/day)	128(47.94)	158(32.11)	**1.78(1.23-2.58)**		1.50(0.94-2.39)	
Women						
Never/Rare drinkers (<3 g/day)	110(78.01)	413(77.06)	1.00(reference)	0.64	1.00(reference)^b^	0.50
Light drinkers (3-20 g/day)	24(17.02)	112(20.07)	0.90(0.55-1.48)		0.87(0.49-1.55)	
Heavy drinkers (≥20 g/day)	7(4.96)	16(2.87)	1.72(0.69-4.31)		0.75(0.27-2.13)	
***H. pylori infection***						
Total						
Negative	38(8.44)	411(39.14)	1.00(reference)		1.00(reference) ^c^	
Positive	412(91.56)	639(60.86)	**6.72(4.69-9.62)**		**7.07(4.60-10.86)**	
Men						
Negative	25(8.42)	168(34.15)	1.00(reference)		1.00(reference) ^c^	
Positive	272(91.58)	324(65.85)	**5.94(3.77-9.37)**		**7.40(4.11-13.31)**	
Women						
Negative	13(8.50)	243(43.55)	1.00(reference)		1.00(reference) ^c^	
Positive	140(91.50)	315(56.45)	**8.13(4.49-14.73)**		**7.54(3.91-14.55)**	
***ALDH2 rs671 genotype*** †						
Total						
GG	815(65.46)	4211(70.86)	1.00(reference)^d^			
AA/AG	430(34.54)	1732(29.14)	**1.23(1.08-1.41)**		NA**	
Men						
GG	518(62.71)	1828(69.56)	1.00(reference)^d^			
AA/AG	308(37.29)	800(30.44)	**1.33(1.13-1.58)**		NA**	
Women						
GG	297(70.88)	2383(71.89)	1.00(reference)^d^			
AA/AG	122 (29.12)	932(28.11)	1.05(0.84-1.31)		NA**	

Abbreviations: OR, Odds Ratio; CI, Confidence Interval;

^a^ Adjusted for age and sex (where appropriate).

^b^ Adjusted for age, sex (where appropriate), smoking status, education level, income, fruit/vegetable intake, *H. pylori* infection status, and family history of gastric cancer.

^c^ Adjusted for age, sex (where appropriate), smoking status, alcohol intake status, education level, income, fruit/vegetable intake, and family history of gastric cancer.

^d^ Adjusted for age, sex (where appropriate), and study sites.

† combined with Korean Genome Epidemiology Study (KoGES) data.

NA**: confounders were auto-adjusted by the randomization effects of genotype.

* p value for trend.

### Interaction between alcohol exposure and *ALDH2* rs671 polymorphism on gastric cancer risk

The estimated gastric cancer risks stratified by the rs671 genotype and alcohol drinking status are displayed in Table 3. An increased risk was observed for the heavy drinkers with the GG genotype (minimally adjusted OR, 2.47; 95% CI, 1.73-3.54), and among the non-heavy drinkers, those with the AA/AG genotype had a 1.34-fold higher risk compared to the GG homozygotes (95% CI, 1.01-1.79), but the OR estimate was not significant in the fully adjusted model. Further analysis of the associations using additional KoGES control subjects revealed that the estimated risks were similar between the heavy drinkers with the AA/AG and GG genotypes (AA/AG group OR, 2.02; 95% CI, 1.16-3.53; GG group OR, 2.47; 95% CI, 1.83-3.34). Similar ORs were also observed between the individuals with the AA/AG genotype in the two drinking groups, and there was no evidence of an interaction between alcohol consumption and the *ALDH2* genotype.

**Table 3:** Association between alcohol consumption behavior and *ALDH2* rs671 polymorphism and gastric cancer risk, with regards to each other

		Case(%)	Control(%)	Minimally Adjusted^a^	Fully Adjusted^b^	P value*
				OR(95% CI)	OR(95% CI)	
***NCC only***						
*ALDH2* genotype AA/AG						
	Never/Rare & Light drinkers (<20 g/day)	117(86.67)	296(94.27)	1.00(reference)	1.00(reference)	0.80
	Heavy drinkers (≥20 g/day)	18(13.33)	18(5.73)	2.01(0.99-4.08)	2.02(0.86-4.77)	
*ALDH2* genotype GG						
	Never/Rare & Light drinkers (<20 g/day)	156(57.14)	580(78.80)	1.00(reference)	1.00(reference)	
	Heavy drinkers (≥20 g/day)	117(42.86)	156(21.20)	**2.47(1.73-3.54)**	**1.59(1.03-2.45)**	
Never/Rare & Light drinkers (<20 g/day)						
	GG	156(57.14)	580(66.21)	1.00(reference)	1.00(reference)	
	AA/AG	117(42.86)	296(33.79)	**1.34(1.01-1.79)**	1.40(0.99-1.98)	
Heavy drinkers (≥20 g/day)						
	GG	117(86.67)	156(89.66)	1.00(reference)	1.00(reference)	
	AA/AG	18(13.33)	18(10.34)	1.36(0.67-2.76)	1.61(0.68-3.82)	
***NCC+KoGES control***						
*ALDH2* genotype AA/AG						
	Never/Rare & Light drinkers (<20 g/day)	117(86.67)	1647(95.09)	1.00(reference)	1.00(reference)^c^	0.99
	Heavy drinkers (≥20 g/day)	18(13.33)	85(4.91)	**2.02(1.16-3.53)**	1.78(0.96-3.29)	
*ALDH2* genotype GG						
	Never/Rare & Light drinkers (<20 g/day)	156(57.14)	3368(79.98)	1.00(reference)	1.00(reference)^c^	
	Heavy drinkers (≥20 g/day)	117(42.86)	843(20.02)	**2.47(1.83-3.34)**	**2.19(1.58-3.03)**	
Never/Rare & Light drinkers (<20 g/day)						
	GG	156(57.14)	3368(67.16)	1.00(reference)	1.00(reference)^c^	
	AA/AG	117(42.86)	1647(32.84)	**1.41(1.10-1.82)**	**1.34(1.02-1.75)**	
Heavy drinkers (≥20 g/day)						
	GG	117(86.67)	843(90.84)	1.00(reference)	1.00(reference)^c^	
	AA/AG	18(13.33)	85(9.16)	1.52(0.89-2.63)	1.28(0.70-2.35)	

Abbreviations: OR, Odds Ratio; CI, Confidence Interval; NCC, National Cancer Center; KoGES, Korean Genome Epidemiology Study;

^a^ Adjusted for age and sex.

^b^ Adjusted for age, sex, smoking status, education level, income, fruit/vegetable intake, *H. pylori* infection status, and family history of gastric cancer.

^c^ Adjusted for age, sex, smoking status, education level, income, family history of gastric cancer, and study sites.

* p value for Cochran-Mantel-Haenszel test.

### Modification of *H. pylori* infection effect on gastric cancer risk by alcohol exposure and *ALDH2* rs671 polymorphism

No significant interaction was detected between *H. pylori* infection and alcohol consumption or rs671 (p for interaction=0.71, 0.23, respectively) (Table 4), and the minimally adjusted ORs for the heavy drinkers in the two *H. pylori* infection status groups were both significant (infection-negative group OR, 2.95; 95% CI, 1.18-7.40; infection-positive group OR, 1.99; 95% CI, 1.44-2.75) compared to those for the non-heavy drinkers. In the fully adjusted model, only the heavy drinkers in the infection-positive group had a significant OR of 1.50 (95% CI, 1.04-2.16). A risk of carrying the A allele was not evident in either infection status group. Atrophic gastritis was considered an additional covariate for the subsamples (n=1,130) in all analyses (Supplementary Table 4). However, it did not have a substantial effect on the estimates of the coefficients or the significance of the interaction, and a problem with overcorrection was observed in association analysis of *H. pylori* infection and the gastric cancer risk (Supplementary Table 5).

**Table 4:** Association between *H. pylori* infection and gastric cancer risk, stratified by alcohol drinking status/*ALDH2* rs671 genotype

		Case(%)	Control(%)	Minimally Adjusted^a^	Fully Adjusted^b^	P value*
				OR(95% CI)	OR(95% CI)	
*H. pylori* infection x Alcohol drinking status						
Negative	Never/Rare & Light drinkers (<20 g/day)	22(62.86)	349(84.91)	1.00(reference)	1.00(reference)	0.35
	Heavy drinkers (≥20 g/day)	13(37.14)	62(15.09)	**2.95(1.18-7.40)**	1.72(0.53-5.58)	
Positive	Never/Rare & Light drinkers (<20 g/day)	251(67.29)	527(82.47)	1.00(reference)	1.00(reference)	
	Heavy drinkers (≥20 g/day)	122(32.71)	112(17.53)	**1.99(1.44-2.75)**	**1.50(1.04-2.16)**	
	P for interaction			0.49	0.71	
*H. pylori* infection x *ALDH2* polymorphism						
Negative	GG	24(63.16)	296(72.02)	1.00(reference)	1.00(reference)	0.33
	AA/AG	14(36.84)	115(27.98)	1.48(0.73-3.02)	1.93(0.83-4.48)	
Positive	GG	280(67.96)	440(78.71)	1.00(reference)	1.00(reference)	
	AA/AG	132(32.04)	199(21.29)	0.96(0.73-1.27)	1.10(0.80-1.51)	
	P for interaction			0.27	0.23	

Abbreviations: OR, Odds Ratio; CI, confidence Interval;

^a^ Adjusted for age and sex.

^b^ Adjusted for age, sex, smoking status, education level, income, fruit/vegetable intake, and family history of gastric cancer.

* p value for Cochran-Mantel-Haenszel test.

## DISCUSSION

This study examined the relationship between alcohol consumption and the gastric cancer risk with regard to the *ALDH2* rs671 polymorphism. The allele distribution was independent of the disease status and other confounding factors, such as the cigarette smoking and socioeconomic statuses, satisfying the assumptions for Mendelian randomization. Because alleles are randomly assigned during gamete formation, their distribution is free from confounders, recall bias, and reverse causation of the disease, and it can reflect the lifetime exposure to the risk factor of interest, allowing us to make precise causal inferences regarding the effect of alcohol consumption on the gastric cancer risk [33, 34].

The heavy drinkers displayed an increased risk of gastric cancer in the minimally adjusted model; however, no significant association was detected after full adjustments for the confounders. Additionally, the results of instrumental variable (IV) analysis using rs671 indicated that an increased level of alcohol consumption among the drinkers was not associated with gastric cancer, which was not consistent with the results of conventional epidemiological analysis (Supplementary Table 6). Alcohol intake is strongly associated with lifestyle factors, such as smoking status, diet, education level, and occupation [13], as confirmed in our sample of more than 5,000 healthy controls (Supplementary Table 3), indicating substantial effects of the confounders on alcohol consumption that could not be fully controlled for by statistical adjustments. Further, the average amount of alcohol consumed markedly differed between the males and females, and alcohol intake did not have any impact on the female participants’ risk of gastric cancer. The small amount of alcohol consumed among the females may be the reason for the lack of association detected between the risk of gastric cancer and alcohol intake or the rs671 genotype; however, there may be an underlying sex-related biological mechanism that should be further examined. These results differ from those of a previous study of Korean populations [17] reporting an OR of 7.24 (2.12-24.70) for female heavy drinkers and the lack of a dose-response association for males. However, this discrepancy between studies could be due to the relatively small number of controls included in the previous study.

A comparison of the genotypes revealed that the individuals with the A allele had a significantly increased risk of gastric cancer compared to that of the GG homozygotes. Considering the effects of randomization of the alleles of the *ALDH2* genotype, the confounders can be considered to have been automatically adjusted in this analysis. Although the G allele was found to be predictive of increased alcohol intake, a protective effect in GG homozygotes compared to A allele carriers against cancer development has also been observed in previous studies [17, 20–23]. These findings have demonstrated that the systematic effect of acetaldehyde is greater than the direct effect of alcohol on the human body as a carcinogen. Additionally, among never drinkers, rs671 A allele carriers have not been reported to have an increased risk in previous studies [17, 20], indicating simultaneous effects of alcohol intake and *ALDH2* polymorphism on each other. Notably, alcohol must enter an individual's body system first before acetaldehyde can be produced by alcohol dehydrogenase and can affect the cancer risk.

There was no evidence of an interaction between alcohol consumption and genotype, and the heavy drinkers in both genotype groups displayed similar effect sizes. Our results differ from those of a Korean study [17] reporting an interaction of the *ALDH2* genotype with alcohol consumption among ‘never’ and ‘ever’ drinkers, as well as those of a Japanese study [20] reporting a significant interaction among groups stratified by the level of drinking. The criteria used to form the alcohol drinking groups in this study are similar to those used in Shin et al.'s study [17]; however, as mentioned above, this previous Korean study used a small number of controls, and the interaction displayed borderline significance, with a p-value of 0.048. On the other hand, Matsuo et al.'s study [20] included a higher proportion of males, which may explain the higher proportion of heavy drinkers in their study population. Additionally, the level of alcohol consumption used to define heavy drinkers, which is the only group with a significant risk in interaction analysis, is much higher than that used in our study. Therefore, the discrepancies between our study and these original studies could be attributed to differences in the composition of the study groups and in stratification of the alcohol consumption groups. This interaction between alcohol consumption and the *ALDH2* genotype has been established to contribute to the risk of esophageal cancer [9, 35], and in the aerodigestive tract, the effects of alcohol on esophageal, stomach, and colorectal cancers are expected to differ [36]. A further study with a larger sample size and appropriate stratification of alcohol consumption must be conducted to fully establish this relationship. However, the importance of distinguishing between the effect of alcohol itself and the secondary carcinogenic effect of acetaldehyde on gastric cancer and of considering both the level of alcohol intake and the rs671 polymorphism are highlighted in this study and in many other studies [17, 20, 37].

*H. pylori* infection was also confirmed to be one of the strongest risk factors for gastric cancer in our study (adjusted OR, 7.07; 95% CI, 4.60-10.86), with a similar effect size compared with those reported in previous studies [38]. Considering that an *ALDH2* polymorphism is an independent risk factor for gastric cancer in East Asians, with a pooled OR of 1.18 [23] and a minor allele frequency (MAF) ranging from 0.13 for the Vietnamese to 0.24 for the Japanese [39], we projected a simple estimation of the gastric cancer incidence based on the *ALDH2* genotype and *H. pylori* infection status, using the lowest gastric cancer incidence rate among Asians of 2.8 for Indonesians as a baseline [40]. A discrepancy between the projected and observed incidence rates [41] was observed (Supplementary Table 7), reflecting the “Asian enigma” of the higher seroprevalences of *H. pylori* infection in the populations of India [42] and Bangladesh [43] but much lower gastric cancer incidence rates compared with East Asians [44–47]. Ma et al. have reported a significant association between heavy alcohol consumption and gastric cancer in Korean gastric cancer patients without *H. pylori* infection [32]. However, in our study, insufficient evidence of an interaction effect between *H. pylori* infection and alcohol on the gastric cancer risk was obtained, which is consistent with the results of a previous Russian study [48]. These differences among the results could be due to the high *H. pylori* infection prevalence for in both the cases and controls in the previous Korean study, in which most of the participants were older farmers.

The current study has several strengths. First, the sample size was relatively large, with inclusion of KoGES data in assessment of the effect of the *ALDH2* genotype. Second, a wide range of potential confounding factors with substantial effects on both the average alcohol intake and the gastric cancer risk, including the *H. pylori* infection status, were considered. In contrast, the smoking and alcohol consumption statuses may have been underreported among the female participants due to cultural norms in Korea. However, when only the *ALDH2* genotype was considered in the risk estimations, no significant association was observed for the females, which actually reflected their low consumption of alcohol. Another limitation is that we were not able to consider the different types of virulence factors for *H. pylori* infection or the different anatomical sites of gastric cancer due to lack of available information. However, the strain types of *H. pylori* were insufficient in explaining the “Asian enigma” or the rapid changes in the pattern of gastric incidence that occur worldwide [28, 31]. Due to a sample size limitation, the drinking groups were combined for analysis, and the risks for the very heavy drinkers and binge drinkers could not be distinguished. Further, some of the non-significant results could have been attributed to the low power of this study, especially considering the lack of epidemiological data for the KoGES case samples.

In summary, we have found that both alcohol consumption and the *ALDH2* rs671 polymorphism must be considered when assessing the gastric cancer risk; however, no interactions of these factors with the gastric cancer risk were observed. *H. pylori* infection was also confirmed to be a strong independent risk factor for gastric cancer among Koreans. However, there was no interacting effect between *H. pylori* infection and alcohol drinking behavior or genetic susceptibility to acetaldehyde metabolism on the gastric cancer risk, and their independent effects were insufficient to explain the discrepancy in risk across Asian countries. Further evaluations of the associations of other environmental factors, such as diet and/or genetic susceptibility, with the risk of *H. pylori* infection are necessary to better understand the etiologic heterogeneity of gastric cancer in Asian populations.

## MATERIALS AND METHODS

### Study population

A total of 450 gastric cancer patients and 1,050 controls who were part of a gastric cancer research project conducted at the National Cancer Center (NCC) in Korea were included in this study. The cases were histologically diagnosed at the Center for Gastric Cancer with early-stage gastric cancer within three months prior to their recruitment between March 2011 and December 2014. The controls were recruited from among individuals who had visited the same hospital during the same time period as beneficiaries of the National Health Insurance program. Control participants with a history of cancer, diabetes mellitus, gastric or duodenal ulcers, or *H. pylori* treatment were excluded. The detailed characteristics of the study participants have been described elsewhere [49, 50], and only individuals with *ALDH2* genotype data were included in this study. All subjects who participated in this study were of Korean origin, and written informed consent was obtained before their enrollment. The study protocol was approved by the Institutional Review Board of the NCC (IRB #: NCCNCS 11-438).

### Data collection

All participants completed a set of self-administered questionnaires on demographics, medical history, and lifestyle, including smoking status and average alcohol intake. The participants were asked whether they had ever consumed an alcoholic beverage during their lifetime, and information on the average number of occasions on which various alcoholic beverages were consumed during the previous year, the average number of drinks consumed on each occasion, and the serving sizes was obtained. The beverage-specific amounts of alcohol consumed were calculated and were then summed to determine the total daily alcohol consumption (g/day). The study subjects were classified according to their daily alcohol intake as never/rare drinkers (<3 g/day or stated that they had never consumed alcohol), light drinkers (3~20 g/day), or heavy drinkers (≥20 g/day). The average alcohol consumption calculated using the questionnaires has been reported to be strongly correlated with the serum γ-glutamyltransferase level in Koreans [51]. Biopsy specimens were obtained from both the greater and lesser curvatures of the antrum and body of the stomach. *H. pylori* infection status was assessed by the rapid urease test (Pronto Dry; Medical Instruments Corporation, Solothurn, Switzerland) and histological evaluations. The degree of gastric atrophy was histologically determined by hematoxylin and eosin staining and by averaging the scores of the obtained biopsy specimens. If the severity grading level determined using the Sydney scoring system [52] was greater than ‘slight’, then the participant was defined as having atrophic gastritis.

Genomic DNA extracted from peripheral blood leukocytes was genotyped using an Affymetrix Axiom®Exom 319 Array (Affymetrix Inc., Santa Clara, CA, USA) with 318,983 SNPs; the details of the quality-control criteria used have been described elsewhere [50]. A SNP in the *ALDH2* gene, rs671, was selected for further analysis; this SNP had a MAF of 0.16 and met Hardy-Weinberg equilibrium in the control samples.

### Korean genome epidemiology study data

A total of 795 gastric cancer patients with data on sex, age, and genotype and 4,893 healthy controls who were recruited as part of the Korean Genome Epidemiology Study (KoGES) from urban (n=3,305) and rural (n=1,588) communities were included to increase the power of the analysis results. Demographic and lifestyle information, including alcohol intake amount, was available only for the controls. Peripheral blood DNA was genotyped using an Affymetrix Genome-wide Human SNP Array 6.0 (Affymetrix Inc., Santa Clara, CA, USA), and the quality control details have been previously described [53]. Because this array did not include rs671, genotype imputation was performed. Pre-phasing of genotypes was performed using SHAPEIT (v2.r837), and imputation was performed using IMPUTE2 (2.3.2). The East Asian Ancestry (EAS) sample of the 1000 Genome Project phase 3 (n=504) was used as a reference panel. The INFO scores for imputed rs671 were all above 0.98 for both the cases and controls.

### Statistical analysis

The general characteristics were compared between the gastric cancer patients and controls using Student's t-test and the chi-square test. Relationships between the rs671 genotypes and potential confounding factors were assessed by analysis of variance and the chi-squared test for the controls only. The distributions of confounding factors among the different drinking groups were also assessed. The risks of alcohol drinking and *H. pylori* infection were analyzed by adjusting for confounders using two models: a minimally adjusted model with age and sex as covariates, and a fully adjusted model with age, sex, smoking status, education level, household income, fruit/vegetable intake, and family history of gastric cancer among first-degree relatives as covariates. *H. pylori* infection status and alcohol intake were also included as covariates where appropriate. Individuals with missing covariate data were not included in analysis. Multivariable logistic regression analysis was performed to evaluate the gastric cancer risk by the *ALDH2* genotype using the combined NCC and KoGES data, with adjustments for sex, age and study sites.

The alcohol drinking status and *ALDH2* genotype were considered together in evaluation of their combined effect on the gastric cancer risk, and the Cochran-Mantel-Haenszel test was performed to check for homogeneity. To increase the study power, the KoGES controls were combined for further analyses. The interaction effect of *H. pylori* infection with alcohol intake and the rs671 polymorphism on the gastric cancer risk was also evaluated. The effect modification was examined using the likelihood ratio test between the models by adding the multiplicative terms of *H. pylori* infection and the *ALDH2* polymorphism or alcohol consumption status to the logistic regression model. The dose-response effect of average daily alcohol intake on gastric cancer was estimated via a Mendelian randomization approach, using rs671 as an IV [54–56] (details in Supplementary Data). All analyses were performed using SAS version 9.3 (SAS Institute Inc., Cary, NC) and Stata 12.0 (Stata Corp, College Station, TX).

## SUPPLEMENTARY MATERIALS FIGURES AND TABLES


