# Supplementary material for: Effects of alcohol consumption, ALDH2 rs671 polymorphism, and Helicobacter pylori infection on the gastric cancer risk in a Korean population
Source: Oncotarget. 2016 Dec 21;8(4):6630–41. doi: 10.18632/oncotarget.14250 (PMC5351658; doi:10.18632/oncotarget.14250)
Supplement: Supplementary file 1 [file oncotarget-08-6630-s001.pdf]

Effects of alcohol consumption, *ALDH2* rs671 polymorphism, and *Helicobacter pylori* infection on the gastric cancer risk in a Korean population

SUPPLEMENTARY DATA

IV analysis method

The average daily alcohol intake was natural log-transformed to correct for skewness. A multivariable logistic regression model with the log-transformed alcohol intake as a continuous independent variable was evaluated to compute the conventional epidemiological ORs, which were adjusted minimally and fully for the same covariates as the previous analysis; only the drinkers with a calculated daily intake were included in the prediction (n=976). The two-stage control function estimator approach [1] was performed, in which the rs671 genotype was used as an IV for alcohol intake. We assumed an additive model, in which an increased number of G allele of rs671 predicted an increased consumption of alcohol. First, linear regression was fitted using only the control participants to predict the amount of alcohol intake using the IV. Second, with the predicted values of alcohol intake and gastric cancer case-control status, logistic regression was performed to estimate the risk of alcohol on gastric cancer and robust standard errors, adjusting for residuals from the first stage. The estimated coefficient from the second stage depicts the effect of IV for alcohol intake on gastric cancer. Both stages of IV analysis were adjusted for age and/or gender. F-statistics from the first-stage regression were used to assess the strength of IV [2], and the Durbin-Wu-Housman [3] statistic was used to compare the conventional analysis and IV analysis estimates. If the null hypothesis of both risk estimates being equal is rejected, this may indicate that there was confounding or bias effect on conventional epidemiological estimates. IV analysis was performed using Stata 12.0 (Stata Corp, College Station, TX).

REFERENCES

1. Palmer TM, Sterne JA, Harbord RM, Lawlor DA, Sheehan NA, Meng S, et al. Instrumental variable estimation of causal risk ratios and causal odds ratios in Mendelian randomization analyses. American Journal of Epidemiology. 2011; 173:1392-403.
2. Staiger DO, Stock JH. Instrumental variables regression with weak instruments. National Bureau of Economic Research Cambridge, Mass., USA; 1994.
3. Baum CF, Schaffer ME, Stillman S. Instrumental variables and GMM: Estimation and testing. Stata Journal. 2003; 3:1-31.
4. Sherry ST, Ward M-H, Kholodov M, Baker J, Phan L, Smigielski EM, et al. dbSNP: the NCBI database of genetic variation. Nucleic Acids Research. 2001; 29:308-11.
5. Ferlay J, Soerjomataram I, Ervik M, Dikshit R, Eser S, Mathers C, et al. GLOBOCAN 2012 v1. 0. Cancer Incidence and Mortality Worldwide: IARC CancerBase. 2013.
6. Helicobacter, Group CC. Gastric cancer and Helicobacter pylori: a combined analysis of 12 case control studies nested within prospective cohorts. Gut. 2001; 49:347-53.
7. Cai Q, Wu J, Cai Q, Chen E-Z, Jiang Z-Y. Association between Glu504Lys Polymorphism of ALDH2 Gene and Cancer Risk: A Meta-Analysis. PloS One. 2015; 10.
8. Lim SH, Kwon J-W, Kim N, Kim GH, Kang JM, Park MJ, et al. Prevalence and risk factors of Helicobacter pylori infection in Korea: nationwide multicenter study over 13 years. BMC gastroenterology. 2013; 13:104.
9. Ueda J, Gosho M, Inui Y, Matsuda T, Sakakibara M, Mabe K, et al. Prevalence of Helicobacter pylori infection by birth year and geographic area in Japan. Helicobacter. 2014; 19:105-10.
10. Zhu Y, Zhou X, Wu J, Su J, Zhang G. Risk factors and prevalence of Helicobacter pylori infection in persistent high incidence area of gastric carcinoma in Yangzhong city. Gastroenterology Research and Practice. 2014; 2014.
11. Hoang TTH, Bengtsson C, Phung DC, Sörberg M, Granström M. Seroprevalence of Helicobacter pylori infection in urban and rural Vietnam. Clinical and Diagnostic Laboratory Immunology. 2005; 12:81-5.
12. Misra V, Misra S, Singh MK, Singh PA, Dwivedi M. Prevalence of H. pylori in patients with gastric cancer. Indian journal of pathology & microbiology. 2007; 50:702-7.
13. Ahmad MM, Rahman M, Rumi AK, Islam S, Hug F, Chowdhury MF, et al. Prevalence of Helicobacter Pylori in Asymptomatic Population a Pilot Serological Study in Bangladesh. Journal of Epidemiology. 1997; 7:251-4.
14. Syam AF, Miftahussurur M, Makmun D, Nusi IA, Zain LH. Risk Factors and Prevalence of Helicobacter pylori in Five Largest Islands of Indonesia: A Preliminary Study. PloS one. 2015; 10.

Supplementary Table 1: Characteristics of the Korean Genome Epidemiology Study (KoGES) subjects, stratified by disease status and sex

	Case (n=795)				Control(n=4893)			
	Male		Female		Male		Female	
	n	Mean ±S.D	n	Mean ±S.D	n	Mean ±S.D	n	Mean ±S.D
Age (years)	529	57.00±10.89	266	54.38±12.82	2136	56.81±8.56	2757	54.68±8.43
ALDH2 genotype (rs671) (%)								
A/A		17(3.21)		3(1.13)		52(2.43)		55(1.99)
A/G		184(34.78)		80(30.08)		583(27.29)		728(26.41)
G/G		328(62.00)		183(68.80)		1501(70.27)		1974(71.60)
Alcohol intake (g/day), only among drinkers					1597	26.04±29.98	710	7.56±14.17
Alcohol drinking status by amount (%)								
Never/Rare drinkers (<3 g/day)						741(34.69)		2408(87.34)
Light drinkers (3-20 g/day)						709(33.19)		281(10.19)
Heavy drinkers (≥20 g/day)						686(32.12)		68(2.417)
Smoking status (%)								
Non-smoker						652(30.52)		2672(96.92)
Ex-smoker						751(35.16)		29(1.05)
Current smoker						733(34.32)		56(2.03)
Education level (%)								
<12 years						878(41.85)		1558(57.30)
≥12 years						1220(58.15)		1161(42.70)
Household income -10,000 KRW/month (%)								
Below 200						962(51.53)		1295(56.26)
200-400						594(31.82)		715(31.06)
Above 400						311(16.66)		292(12.68)
Family history of gastric cancer in 1st degree relatives (%)								
Yes						148(6.97)		208(7.59)
No						1976(93.03)		2531(92.41)

Abbreviations: S.D, Standard Deviation; KRW, South Korean Won;

Supplementary Table 2: Distribution of confounding variables stratified by the *ALDH2* rs671 genotype in combined control samples

	n	A/A	n	A/G	n	G/G	P value[†]
		Mean ±S.D		Mean ±S.D		Mean ±S.D	
Age (years)	129	23.84(2.98)	1603	24.09(3.03)	4211	24.15(3.05)	0.41
Sex							0.13
Male		62(48.06)		738(46.04)		1828(43.41)	
Female		67(51.94)		865(53.96)		2383(56.59)	
Alcohol intake (g/day), only among drinkers	7	7.29(7.53)	466	11.64(17.40)	2521	20.43(27.14)	<.0001
Alcohol drinking Status by amount (%)							<.0001
Never/Rare drinkers (<3 g/day)		122(94.57)		1140(71.12)		1697(40.30)	
Light drinkers (3-20 g/day)		0		11(0.69)		48(1.14)	
Heavy drinkers (≥20 g/day)		7(5.43)		452(28.20)		2466(58.56)	
Smoking status (%)							0.07
Non-smoker		90(69.77)		1028(64.13)		2820(66.97)	
Ex-smoker		15(11.63)		289(18.03)		738(17.53)	
Current smoker		24(18.60)		286(17.84)		653(15.51)	
Education level (%)							0.12
<12 years		48(37.80)		670(43.00)		1859(45.08)	
≥12 years		79(62.20)		888(57.00)		2265(54.92)	
Household income -10,000 KRW/ month (%)							0.10
Below 200		49(42.98)		626(45.53)		1757(48.66)	
200-400		39(34.21)		472(34.33)		1222(33.84)	
Above 400		26(22.81)		277(20.15)		632(17.50)	
Fruit and vegetable intake (%)[a,b,*]							
Lowest tertile (<426.55 g/day)		7(31.82)		88(30.66)		249(34.58)	0.80
Middle tertile (<649.47 g/day)		8(36.36)		97(33.80)		236(32.78)	
Highest tertile (≥649.47 g/day)		7(31.82)		102(35.54)		235(32.64)	
H. pylori infection status (%)*							0.21
Positive		17(77.27)		182(62.33)		440(59.78)	
Negative		5(22.73)		110(37.67)		296(40.22)	
Family history of gastric cancer in 1st degree relatives (%)							0.78
Yes		10(7.75)		140(8.76)		344(8.22)	
No		119(92.25)		1458(91.24)		3840(91.78)	

Abbreviations: S.D, Standard Deviation; KRW, South Korean Won;
[a] Adjusted for energy intake, [b] Tertile divided among controls.
* Data available for only National Cancer Center (NCC) controls.
[†] p-values obtained by analysis of variance test and chi-squared test.

Supplementary Table 3: Distribution of confounding variables stratified by the alcohol intake status in combined control samples

	Never/Rare drinkers		Light drinkers		Heavy drinkers		P value†
	n	Mean ±S.D	n	Mean ±S.D	n	Mean ±S.D	
Age (years)	3727	55.30±8.60	1288	53.91±8.78	928	55.17±8.58	<0.0001
Sex							<0.0001
Male		889(23.85)		895(69.49)		844(90.95)	
Female		2838(76.15)		393(30.51)		84(9.05)	
Alcohol intake (g/day), only among drinkers	778	1.42±0.79	1288	9.28±4.83	928	47.34±31.01	<0.0001
Smoking status (%)							<0.0001
Non-smoker		3127(83.90)		589(45.73)		222(23.92)	
Ex-smoker		320(8.59)		365(28.34)		357(38.47)	
Current smoker		280(7.51)		334(25.93)		349(37.61)	
Education level (%)							<0.0001
<12 years		1777(48.64)		409(32.80)		391(43.01)	
≥12 years		1876(51.36)		838(67.20)		518(56.99)	
Household income -10,000 KRW/ month (%)							
Below 200		1629(51.47)		418(37.09)		386(47.65)	<0.0001
200-400		1014(32.04)		438(38.86)		281(34.78)	
Above 400		522(16.49)		271(24.05)		142(17.57)	
Fruit and vegetable intake (%)[a,b,*]							<0.0001
Lowest tertile (<426.55 g/day)		146(25.84)		109(37.20)		89(52.05)	
Middle tertile (<649.47 g/day)		207(36.64)		82(27.99)		52(30.41)	
Highest tertile (≥649.47 g/day)		212(37.52)		102(34.81)		30(17.54)	
H. pylori infection status (%) *							0.06
Positive		333(57.61)		194(65.10)		112(64.37)	
Negative		245(42.39)		104(34.90)		62(35.63)	
Family history of gastric cancer in 1st degree relatives (%)							0.94
Yes		313(8.44)		104(8.13)		77(8.35)	
No		3396(91.56)		1176(91.88)		845(91.65)	

Abbreviations: S.D, Standard Deviation; KRW, South Korean Won;
[a] Adjusted for energy intake, [b] Tertile divided among controls.
* Data available for only National Cancer Center (NCC) controls.
† p-values obtained by analysis of variance test and chi-squared test.

Supplementary Table 4: Distribution of atrophic gastritis status in subsamples (n=1,130) of National Cancer Center (NCC) participants

	Total		
	**Case (%)**	**Control (%)**	**P value†**
Gastric atrophy			<0.0001
Present	283(66.75)	141(19.97)	
Absent	141(33.25)	565(80.03)	

† obtained by chi-squared test.

Supplementary Table 5: Associations of alcohol conusmption, *ALDH2* rs671 polymorphsim, and *H. pylori* infection, with further adjusment for atrophic gastritis

Table #			Fully adjusted OR(95% CI)	P value*
Table 2	***Alcohol intake***			
	Total			
	Never/Rare drinkers (<3 g/day)		1.00(reference)[a]	0.746
	Light drinkers (3-20 g/day)		0.74(0.48-1.13)	
	Heavy drinkers (≥20 g/day)		1.12(0.71-1.77)	
	Men			
	Never/Rare drinkers (<3 g/day)		1.00(reference)[a]	0.242
	Light drinkers (3-20 g/day)		0.86(0.48-1.54)	
	Heavy drinkers (≥20 g/day)		1.30(0.74-2.30)	
	Women			
	Never/Rare drinkers (<3 g/day)		1.00(reference)[a]	0.290
	Light drinkers (3-20 g/day)		0.70(0.37-1.34)	
	Heavy drinkers (≥20 g/day)		0.73(0.24-2.16)	
	H. pylori infection			
	Total			
	Negative		1.00(reference)[b]	
	Positive		**3.97(2.47-6.40)**	
	Men			
	Negative		1.00(reference)[b]	
	Positive		**3.34(1.69-6.60)**	
	Women			
	Negative		1.00(reference)[b]	
	Positive		5.52(2.69-11.34)	
Table 3	*ALDH2* genotype AA/AG			
		Never/Rare & Light drinkers (<20 g/day)	1.00(reference)[a]	
		Heavy drinkers (≥20 g/day)	2.24(0.82-6.15)	
	ALDH2 genotype GG			
		Never/Rare & Light drinkers (<20 g/day)	1.00(reference)[a]	
		Heavy drinkers (≥20 g/day)	1.35(0.83-2.20)	
Table 4	*H. pylori* infection x Alcohol drinking status			
	Negative	Never/Rare & Light drinkers (<20 g/day)	1.00(reference)[c]	
		Heavy drinkers (≥20 g/day)	1.26(0.29-5.44)	
	Positive	Never/Rare & Light drinkers (<20 g/day)	1.00(reference)[c]	
		Heavy drinkers (≥20 g/day)	1.26(0.83-1.92)	
		P for interaction	0.357	
	H. pylori infection x *ALDH2* polymorphism			
	Negative	GG	1.00(reference)[c]	
		AA/AG	1.92(0.73-5.01)	
	Positive	GG	1.00(reference)[c]	
		AA/AG	1.22(0.85-1.75)	
		P for interaction	0.433	

a Adjusted for age, sex (where appropriate), smoking status, education level, income, fruit/vegetable intake, *H. pylori* infection status, family history of gastric cancer, and gastric atrophy status.
b Adjusted for age, sex (where appropriate), smoking status, alcohol intake status, education level, income, fruit/vegetable intake, family history of gastric cancer, and gastric atrophy status.
c Adjusted for age, sex, smoking status, education level, income, fruit/vegetable intake, family history of gastric cancer, and gastric atrophy status.
* p value for trend.

Supplementary Table 6: Estimates of effect of alcohol intake (g/day, log transformed) on gastric cancer risk, comparing conventional and IV Analysis, stratified by sex and *H.pylori* infection status

	Conventional analysis		Instrumental variable analysis		Comparison of the analyses
	Minimally adjusted OR(95% CI) [a]	Fully adjusted OR(95% CI) [b]	F statistics	IV-OR(95% CI)	Durbin-Wu-Housman p
All	**1.21(1.09-1.36)**	1.07(0.94-1.21)	103.25	0.90(0.63-1.28)	0.08
Male	**1.27(1.12-1.45)**	1.15(0.99-1.34)	35.91	0.90(0.63-1.27)	**0.03**
Female	1.08(0.87-1.34)	0.95(0.75-1.19)	5.33	1.07(0.31-3.72)	0.99
H. pylori positive	**1.21(1.07-1.37)**	1.07(0.93-1.22) [c]	61.63	0.99(0.69-1.42)	0.25
H. pylori negative	1.14(0.82-1.59)	1.03(0.71-1.50) [c]	42.57	0.54(0.17-1.66)	0.20

Abbreviations: OR, Odds Ratio; CI, Confidence Interval; IV, Instrumental Variable.

[a] Adjusted for age and sex (where appropriate).

[b] Adjusted for age, sex (where appropriate), smoking status, education level, income, fruit/vegetable intake, *H. pylori* infection status, and family history of gastric cancer.

c Adjusted for age, sex, smoking status, education level, income, fruit/vegetable intake, and family history of gastric cancer.

Supplementary Table 7: Gastric cancer incidence in Asia (2012) and projected incidence using *H. pylori* infection and *ALDH2* rs671 polymorphism

Region	*H.pylori* infection seroprevalence (%)	*ALDH2* rs671 polymorphism MAF [4]	Observed gastric cancer ASR (W) (per 100,000) [5]	Projected gastric cancer incidence rate (per 100,000) *
	RR=5.9 [6]	Pooled OR=1.18 [7]		Additive effect of *H. pylori* and rs671
Korea	54.4 [8]	16.7	41.8	7.6
Japan	39.9 [9]	24	29.9	5.7
China	63.4 [10]	16.0~27.1	22.7	8.9
Vietnam	74.6 [11]	13.6	16.3	10.4
India	80.0 [12]	0	6.1	11.0
Bangladesh	92.0 [13]	0	5.7	12.6

Abbreviations: RR, Relative Risk; OR, Odds Ratio; MAF, Minor Allele Frequency; ASR (W), Age-world-standardized incidence rate;

* Indonesia's ASR of 2.8 [14] was used as a baseline incidence rate among Asian countries.
